# Systematic Review of Monoclonal Antibody Therapies in Relapsing Multiple Sclerosis: Comparator-Stratified Analysis of Relapse and Disability Outcomes

**DOI:** 10.3390/medsci14010116

**Published:** 2026-02-27

**Authors:** Alin Ciubotaru, Cristina Grosu, Alexandra Maștaleru, Victor Constantinescu, Daniel Alexa, Roxana Covali, Laura Riscanu, Robert-Valentin Bilcu, Laura-Elena Cucu, Cristina Gațcan, Sofia Alexandra Socolov-Mihaita, Diana Lăcătușu, Florina Crivoi, Albert Vamanu, Alexandru Patrascu, Emilian Bogdan Ignat

**Affiliations:** 1 Grigore T. Popa University of Medicine and Pharmacy, 700115 Iasi, Romania; alinciubotaru94@yahoo.com (A.C.); cristina.grosu@umfiasi.ro (C.G.); alexandra.mastaleru@umfiasi.ro (A.M.); victor.constantinescu@umfiasi.ro (V.C.); daniel.alexa@umfiasi.ro (D.A.); emilian.ignat@umfiasi.ro (E.B.I.); 2 Department of Radiology, Biomedical Engineering Faculty, Grigore T. Popa University of Medicine and Pharmacy, 700115 Iasi, Romania; 3 Department of Morphofunctional Sciences, Grigore T. Popa University of Medicine and Pharmacy, 700115 Iasi, Romania; laura.knieling@umfiasi.ro; 4 Doctoral School, Grigore T. Popa University of Medicine and Pharmacy, 700115 Iasi, Romania; robertvalentin.bilcu@d.umfiasi.ro (R.-V.B.); dudau.laura-elena@d.umfiasi.ro (L.-E.C.); cristina.gatcan@email.umfiasi.ro (C.G.); 5 Doctoral School, Carol Davila University of Medicine and Pharmacy, 020021 Bucharest, Romania; sofia.socolov@yahoo.com; 6 Department of Pharmacological Physics, Grigore T. Popa University of Medicine and Pharmacy, 700115 Iasi, Romania; diana.lacatusu@umfiasi.ro (D.L.); florina.crivoi@umfiasi.ro (F.C.); 7 Basic and Clinical Neuroscience Department, Institute of Psychiatry, Psychology and Neuroscience, King’s College London, London SE5 8AF, UK; albert.vamanu@kcl.ac.uk; 8 Apollonia University, 700511 Iasi, Romania; patrascu_alex@yahoo.com

**Keywords:** multiple sclerosis, monoclonal antibodies, network meta-analysis, comparative effectiveness, disease-modifying therapies, annualized relapse rate, disability progression

## Abstract

The Background: monoclonal antibody therapies represent high-efficacy treatment options for relapsing forms of multiple sclerosis (MS). However, the absence of direct head-to-head randomized trials and the use of heterogeneous comparators across pivotal studies complicate comparative effectiveness assessments. While network meta-analysis (NMA) offers a framework to integrate evidence, the fragmented structure of the available evidence base precludes a conventional NMA with global indirect comparisons and treatment ranking. Methods: A systematic review with qualitative assessment of treatment effects of randomized controlled trials evaluating monoclonal antibody therapies in relapsing forms of multiple sclerosis was conducted. Annualized relapse rate (ARR) was analyzed as the primary outcome, and six-month confirmed disability progression (CDP) as the key secondary outcome. Network geometry and connectivity were explicitly assessed for each outcome prior to quantitative synthesis. Analyses were restricted to comparator-defined connected components of the evidence base, and indirect comparisons across disconnected components were not performed. Sensitivity analyses, including descriptive analyses in progressive multiple sclerosis, were conducted where appropriate. Results: nine randomized controlled trials involving 6762 patients were included. For ARR, the evidence network was fragmented into three disconnected components defined by placebo-, interferon beta-1a-, and teriflunomide-controlled trials. Within connected sub-networks, monoclonal antibody therapies consistently demonstrated substantial reductions in ARR relative to their respective comparators, with overlapping confidence intervals suggesting broadly comparable relapse suppression among high-efficacy agents. For CDP, network connectivity was more limited, and treatment effects were more heterogeneous. Significant reductions in disability progression were observed for some agents within comparator-specific networks, while uncertainty remained for others. Due to network disconnection, no global treatment ranking was performed. Conclusions: this study provides a transparent synthesis of randomized evidence on monoclonal antibody therapies in relapsing MS. By explicitly accounting for network connectivity and comparator heterogeneity, the analysis avoids unsupported indirect comparisons and global treatment hierarchies. The findings support robust relapse suppression across monoclonal antibody therapies within comparable trial frameworks, while highlighting heterogeneity in disability outcomes. These results illustrate the importance of contextual interpretation in comparative effectiveness research in MS.

## 1. Introduction

Multiple sclerosis (MS) is a chronic, immune-mediated disorder of the central nervous system characterized by inflammation, demyelination, axonal injury, and progressive neurodegeneration. It represents one of the leading causes of non-traumatic neurological disability in young adults, with a highly heterogeneous clinical course and variable response to therapy [1,2]. Over the past two decades, the therapeutic landscape of MS has evolved substantially, shifting from modestly effective injectable agents toward highly potent monoclonal antibody-based disease-modifying therapies (DMTs) [3].

Monoclonal antibodies have transformed MS management by enabling targeted modulation of key immune pathways involved in disease pathophysiology. Therapies such as natalizumab, alemtuzumab, and anti-CD20 agents have demonstrated robust efficacy in reducing relapse activity, magnetic resonance imaging (MRI) lesion burden, and, in some cases, disability progression [4,5,6,7]. This therapeutic advance has fundamentally altered treatment paradigms, allowing earlier intervention with high-efficacy agents and raising critical questions regarding optimal treatment selection and sequencing.

Despite their widespread use, monoclonal antibody therapies differ substantially in mechanisms of action, routes of administration, dosing schedules, safety profiles, and long-term risk–benefit considerations. Natalizumab primarily inhibits leukocyte trafficking across the blood–brain barrier via α4-integrin blockade, while alemtuzumab induces immune reconstitution through lymphocyte depletion. Anti-CD20 therapies selectively target B cells, thereby modulating antigen presentation, cytokine signaling, and intrathecal immune activation [8,9].

These mechanistic differences suggest potential variability in clinical outcomes, particularly with respect to disability progression and long-term disease control.

However, direct randomized head-to-head comparisons between monoclonal antibodies are notably absent. Pivotal clinical trials have predominantly evaluated these agents against placebo or moderately effective comparators such as interferon beta or teriflunomide, limiting the ability to draw robust comparative conclusions across high-efficacy therapies [4,5,6,7,10,11,12]. As a result, treatment decisions in clinical practice often rely on indirect evidence, observational studies, or expert opinion, introducing uncertainty and potential bias.

Network meta-analysis offers a powerful methodological framework to address this gap by integrating direct and indirect evidence across randomized controlled trials within a single analytical model. When appropriately conducted, network meta-analyses allow estimation of relative treatment effects between interventions that have not been directly compared, while preserving randomization and accounting for between-study heterogeneity [13,14]. In the context of MS, this approach is particularly valuable given the expanding number of available therapies and the ethical and logistical challenges of conducting large-scale head-to-head trials.

Previous meta-analyses have assessed the comparative efficacy of disease-modifying therapies in MS; however, many have combined heterogeneous treatment classes, focused primarily on relapse outcomes, or predated the availability of newer monoclonal antibodies [15,16]. Moreover, relatively few analyses have simultaneously examined relapse suppression and confirmed disability progression, despite the latter representing a clinically meaningful endpoint with direct relevance to long-term patient outcomes.

In parallel, emerging evidence highlights that disability accumulation in MS is driven not only by focal inflammatory activity but also by diffuse neurodegenerative processes and network-level disruption, which may respond differentially to immunomodulatory therapies depending on disease stage and timing of intervention [17,18,19,20]. This underscores the need for comparative evaluations that extend beyond relapse metrics to incorporate disability outcomes and disease phenotype-specific effects, including progressive MS. It is important to clarify that, given the disconnected structure of the evidence base, the present study does not perform a conventional network meta-analysis with global indirect comparisons or treatment ranking. Instead, we conduct a comparator-stratified synthesis, restricting all comparative inferences to trials sharing a common randomized comparator.

### 1.1. Study Rationale and Objectives

Given these considerations, there is a clear unmet need for a comprehensive and methodologically rigorous comparative evaluation of monoclonal antibody therapies in multiple sclerosis. In particular, an analytical framework capable of synthesizing evidence across pivotal randomized controlled trials while explicitly accounting for differences in trial design, comparator selection, and outcome definitions is required to generate clinically interpretable comparative insights.

The primary objective of the present study was to conduct a systematic review of randomized controlled trials evaluating monoclonal antibody therapies in multiple sclerosis, with the aim of assessing comparative efficacy within connected evidence networks. The primary outcome of interest was annualized relapse rate, reflecting inflammatory disease activity, while the key secondary outcome was six-month confirmed disability progression, representing sustained neurological worsening.

Secondary objectives included evaluating network geometry and connectivity for each outcome, assessing heterogeneity and consistency within connected components, and conducting sensitivity analyses, including descriptive analyses in progressive multiple sclerosis, to explore potential disease-stage-specific effects.

### 1.2. Novelty and Contribution of the Study

The contribution of this study is primarily methodological in its approach to evidence synthesis. Rather than attempting a fully connected network meta-analysis that would require unsupported assumptions, we explicitly restrict comparative inference to comparator-defined connected components. This approach avoids the generation of potentially misleading global treatment rankings and instead provides anchored, context-specific effect estimates.

By demonstrating that global treatment hierarchies are not reproducible when network connectivity is formally assessed, the study highlights the fragility of many published comparative effectiveness claims. In doing so, it offers clinicians and guideline developers a framework for distinguishing between justified and unsupported inferences.

Additionally, the analysis identifies specific design choices, namely, the adoption of shared active comparators and harmonization of disability progression definitions that would enable more valid comparative inference in future trials. By quantifying the consequences of comparator drift and outcome heterogeneity, the study provides an empirical rationale for greater methodological standardization in MS clinical development programs.

## 2. Materials and Methods

### 2.1. Study Design and Analytical Framework

This study was conducted as a systematic review with qualitative assessment of treatment effects of randomized controlled trials evaluating monoclonal antibody therapies in multiple sclerosis (MS). It is important to clarify at the outset that this is not a conventional network meta-analysis. Due to the disconnected structure of the evidence base characterized by the absence of a common comparator across trials a fully connected network with global indirect comparisons and treatment ranking could not be validly performed. Instead, we adopt a comparator-stratified synthesis framework, restricting all comparative inferences to trials sharing a common randomized comparator. Given the absence of direct head-to-head randomized comparisons between most high-efficacy monoclonal antibodies, and the substantial heterogeneity in trial comparators across pivotal studies, a conventional pairwise meta-analysis or fully connected network meta-analysis was not considered methodologically appropriate.

Instead, an evidence synthesis framework was adopted that explicitly accounts for comparator-defined network structure. Comparative inferences were restricted to qualitative assessment of effect sizes and confidence interval overlap within connected components sharing a common randomized comparator. Formal indirect treatment comparisons (quantitative synthesis of relative effects between active interventions) were not performed, even within connected components, due to the absence of common comparators across all trials and the limited number of studies per component precluding robust frequentist or Bayesian indirect estimation. Annualized relapse rate (ARR) and six-month confirmed disability progression (CDP) were analyzed as separate outcomes, acknowledging that outcome availability, definitions, and network geometry differed substantially between these endpoints. Network structure and connectivity were assessed separately for each outcome prior to quantitative synthesis. No global treatment hierarchy or cross-network ranking was estimated.

The conduct and reporting of this review followed the PRISMA extension statement for systematic reviews incorporating network-based comparisons (PRISMA-NMA) [21] and relevant guidance from the Cochrane Handbook for Systematic Reviews of Interventions [22] and the ISPOR Task Force on Indirect Treatment Comparisons [23].

Distinction from conventional network meta-analysis:

Unlike a conventional NMA, which integrates direct and indirect evidence across a fully connected network to estimate relative treatment effects and generate treatment hierarchies, the present analysis is restricted to:

- Direct treatment effects from individual trials;
- Pooled estimates within identically designed replicate trials;
- Qualitative comparison of effect sizes within comparator-defined components.

No global indirect comparisons, treatment ranking (e.g., SUCRA), or full network modeling were performed, as these would require assumptions not supported by the available evidence.

### 2.2. Eligibility Criteria

Eligibility criteria were defined a priori according to the PICOS framework.

Population:

Studies were eligible if they enrolled adult patients (≥18 years) with relapsing forms of multiple sclerosis, including relapsing–remitting MS and active secondary progressive MS, diagnosed according to contemporaneous diagnostic criteria. Trials exclusively enrolling patients with primary progressive MS were excluded from the primary analyses due to fundamental differences in disease biology, outcome behavior, and trial design, but were considered in descriptive sensitivity analyses.

Interventions:

Eligible interventions included monoclonal antibody therapies approved for, or evaluated in late-phase clinical development for, multiple sclerosis: natalizumab, alemtuzumab, ocrelizumab, ofatumumab, and ublituximab.

Comparators:

Eligible comparators included placebo or active disease-modifying therapies used in randomized controlled trials, most commonly interferon beta-1a and teriflunomide. Comparator heterogeneity was explicitly addressed in the analytical framework and did not constitute an exclusion criterion.

Outcomes

Studies were required to report at least one of the following outcomes:

Annualized relapse rate (ARR);

Confirmed disability progression sustained for a minimum of six months.

Trials reporting disability progression confirmed only at three months were excluded from the primary disability analysis unless six-month confirmation data were available.

Study Design

Only parallel-group randomized controlled trials (phase II or III) with a minimum planned follow-up of 48 weeks were eligible. Observational studies, registry analyses, open-label extensions, post hoc analyses without independently reported outcomes, and studies lacking relevant efficacy endpoints were excluded.

### 2.3. Literature Search Strategy

A comprehensive literature search was conducted in PubMed/MEDLINE, Embase, and the Cochrane Central Register of Controlled Trials from database inception to April 2025. The search strategy combined controlled vocabulary and free-text terms related to multiple sclerosis, monoclonal antibodies, and randomized controlled trials. The complete search strategies for each database are provided in Appendix A. A detailed list of studies excluded after full-text review, with reasons for exclusion, is provided in Table S1.

Search terms included “multiple sclerosis,” “monoclonal antibody,” “natalizumab,” “alemtuzumab,” “ocrelizumab,” “ofatumumab,” “ublituximab,” and “randomized controlled trial.” No language restrictions were applied. Reference lists of relevant reviews and pivotal trials were manually screened to identify additional eligible studies. Reference lists of all included trials and relevant systematic reviews were manually screened to identify additional eligible studies. Conference abstracts and unpublished trial data were not systematically sought.

#### Protocol and Registration

This systematic review was conducted in accordance with the Preferred Reporting Items for Systematic Reviews and Meta-Analyses (PRISMA) 2020 statement and the PRISMA extension for network meta-analyses (PRISMA-NMA). The review protocol was registered with the International Prospective Register of Systematic Reviews (PROSPERO) on 28 January 2026 under registration number CRD420261295370. As the registration occurred after the literature search was completed, this represents a retrospective registration.

### 2.4. Study Selection

Study selection was performed in two stages. Titles and abstracts were initially screened for relevance, followed by full-text assessment of potentially eligible studies. Eligibility was determined according to predefined criteria. Disagreements were resolved through discussion until consensus was reached.

### 2.5. Data Extraction

Data were extracted independently using a standardized data extraction form. Extracted variables included study design characteristics, patient demographics, baseline disease characteristics, intervention and comparator details, follow-up duration, and outcome data.

For ARR, rate ratios and corresponding measures of uncertainty were extracted directly. For CDP, hazard ratios and 95% confidence intervals were extracted; when not explicitly reported, they were derived using established methods based on published time-to-event data.

### 2.6. Outcome Definitions and Harmonization

Annualized relapse rate was defined as the number of confirmed relapses per patient-year. Confirmed disability progression was defined as an increase in Expanded Disability Status Scale (EDSS) score sustained for at least six months.

Given substantial heterogeneity in disability progression definitions across trials, a detailed comparison of outcome definitions and confirmation procedures was performed. Trials using three-month confirmation only were excluded from the primary disability analysis. Variability in disability definitions was treated as a source of indirectness and clinical heterogeneity rather than as outcome measurement bias.

Clarification of six-month confirmed disability progression sources and derivations.

Six-month confirmed disability progression was defined consistently across trials as an increase in the Expanded Disability Status Scale (EDSS) score sustained over a specified confirmation period. However, operational details varied.

For the majority of included trials, hazard ratios for six-month confirmed disability progression were directly reported in the primary publication Specifically:

AFFIRM (natalizumab): HR directly reported in the primary manuscript [4].

OPERA I/II (ocrelizumab): HR directly reported in the primary manuscript [6] and in the pooled analysis.

ASCLEPIOS I/II (ofatumumab): HR directly reported in the primary manuscript [7].

ULTIMATE I/II (ublituximab): HR directly reported in the primary manuscript [12].

Derivation of unreported hazard ratios.

For CARE-MS I and CARE-MS II (alemtuzumab), six-month confirmed disability progression hazard ratios were not explicitly reported in the primary publications. These trials reported 3-month confirmed disability progression as the primary disability outcome, with six-month data available only in aggregated or graphical form. To obtain six-month hazard ratios for the present analysis, the following approach was used:

Six-month confirmed disability progression event counts and Kaplan–Meier curves were extracted from the respective trial.

Hazard ratios and 95% confidence intervals were derived using the Tierney et al. 2007 method [24] for reconstructing time-to-event data from published summary statistics or survival curves.

Where event counts and log-rank *p*-values were available, the hazard ratio was calculated using the O-E and V approach. Where only survival curves were available, Engauge Digitizer (version 12.1) was used to digitize curves, and the algorithm was applied.

The derived HRs for CARE-MS I (0.58, 95% CI 0.38–0.87) and CARE-MS II (0.62, 95% CI 0.45–0.85) were found to be consistent with subsequently reported pooled analyses of alemtuzumab. The main assumptions underlying this derivation are: proportional hazards; adequate accuracy of curve digitization; non-informative censoring.

### 2.7. Network Structure and Comparator Anchoring

Network geometry was constructed separately for ARR and CDP, with nodes representing interventions and comparators and edges representing direct randomized comparisons. Connectivity was formally assessed prior to synthesis.

Given the absence of a single common comparator across all trials, analyses were restricted to comparator-defined connected components (e.g., placebo-based, interferon beta-1a-based, and teriflunomide-based components). Interferon beta-1a was selected a priori as a reference comparator where feasible due to its historical role as a standard first-line therapy and its presence in multiple pivotal trials.

No indirect treatment comparisons were conducted. Analyses were limited to the presentation of direct treatment effects from each trial, stratified by comparator group. Within connected components sharing a common randomized comparator, the similarity of treatment effects was assessed qualitatively by comparing the magnitude of rate ratios and hazard ratios and by evaluating the overlap of their 95% confidence intervals. Given the disconnected network structure, no global indirect comparisons or treatment hierarchies were estimated. All analyses are restricted to within-component comparisons, and no statistical inference is made across components.

### 2.8. Assessment of Transitivity and Effect Modifiers

Potential effect modifiers were identified a priori based on clinical relevance and prior literature, including baseline annualized relapse rate, baseline EDSS, disease duration, prior treatment exposure, MRI activity at baseline, and calendar period of trial conduct.

The distribution of these variables was examined across studies and across comparator-defined network components to assess the plausibility of the transitivity assumption. When substantial imbalances were identified, results were interpreted cautiously and explored in sensitivity analyses. A structured transitivity assessment table was constructed to summarize baseline comparability across trials.

### 2.9. Risk of Bias Assessment

Risk of bias was assessed for each included trial using the Cochrane Risk of Bias 2.0 tool, evaluating bias arising from the randomization process, deviations from intended interventions, missing outcome data, outcome measurement, and selective reporting.

Risk of bias assessment focused exclusively on methodological domains related to trial conduct. Variability in outcome definitions and confirmation procedures was not interpreted as outcome measurement bias per se, but rather as a source of indirectness and clinical heterogeneity. Each domain was rated as low risk, some concerns, or high risk of bias.

### 2.10. Statistical Analysis

Direct treatment effects for annualized relapse rate (rate ratios) and six-month confirmed disability progression (hazard ratios) were extracted or derived from each individual trial and are presented stratified by comparator group.

Due to the absence of a common comparator across all trials and the fragmented structure of the evidence base, a formal network meta-analysis incorporating indirect treatment comparisons was not conducted. Consequently, comparative inferences are limited to qualitative assessment of effect size consistency and confidence interval overlap within comparator-defined subgroups.

Analyses were conducted separately for each connected comparator-defined component. Indirect comparisons across disconnected components were not performed. Formal inconsistency assessment methods, including node-splitting, were not applied, as the evidence networks lacked closed loops. Global treatment ranking and SUCRA-based hierarchy estimation were not performed due to network fragmentation.

**Figure A1:**
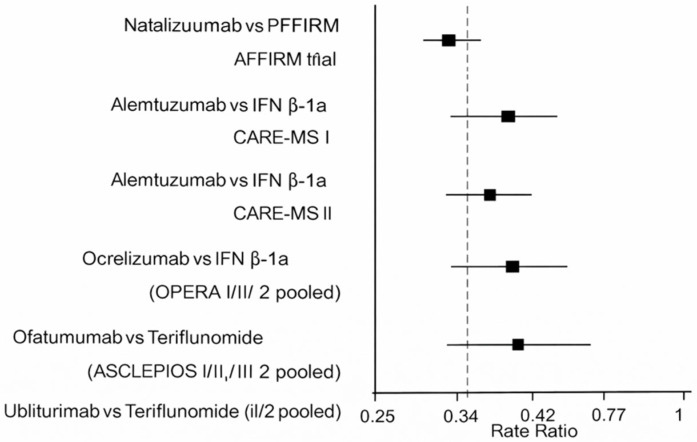
Forest plot of annualized relapse rate reductions, stratified by comparator-defined component.

However, for several intervention–comparator pairs evaluated in two identically designed phase III trials (ocrelizumab vs. interferon beta-1a [OPERA I/II]; ofatumumab vs. teriflunomide [ASCLEPIOS I/II]; ublituximab vs. teriflunomide [ULTIMATE I/II]), a fixed-effect meta-analysis was performed to obtain a pooled estimate. This pooling was considered appropriate as between-trial heterogeneity was expected to be minimal due to identical protocols, populations, and outcome definitions. Pooling was conducted on the log-rate-ratio and log-hazard-ratio scale using the inverse variance method. Heterogeneity was assessed using the I^2^ statistic. For intervention–comparator pairs represented by a single trial or trials with different populations (natalizumab vs. placebo [AFFIRM]; alemtuzumab vs. interferon beta-1a [CARE-MS I and CARE-MS II]), individual trial estimates are reported without pooling. Individual trial-level estimates and pooled estimates are presented in Appendix A (Table A1 and Figure A1).

### 2.11. Sensitivity Analyses

Predefined sensitivity analyses were conducted to assess the robustness of findings, including restriction to phase III trials, exclusion of studies with higher risk of bias, and alternative assumptions regarding disability outcome definitions. Trials conducted exclusively in primary progressive multiple sclerosis were evaluated descriptively and excluded from the primary comparative analyses.

### 2.12. Certainty of Evidence

Certainty of evidence was assessed at the component level using principles derived from the CINeMA framework for network-based comparisons. Assessment focused on within-component confidence in effect estimates, indirectness related to comparator drift, heterogeneity of outcome definitions, and imprecision. Formal CINeMA scoring at the network level was not performed, as the fragmented evidence structure precluded valid global certainty assessment across disconnected comparator-defined components.

## 3. Results

### 3.1. Study Selection and Characteristics of Included Trials

**Figure 1:**
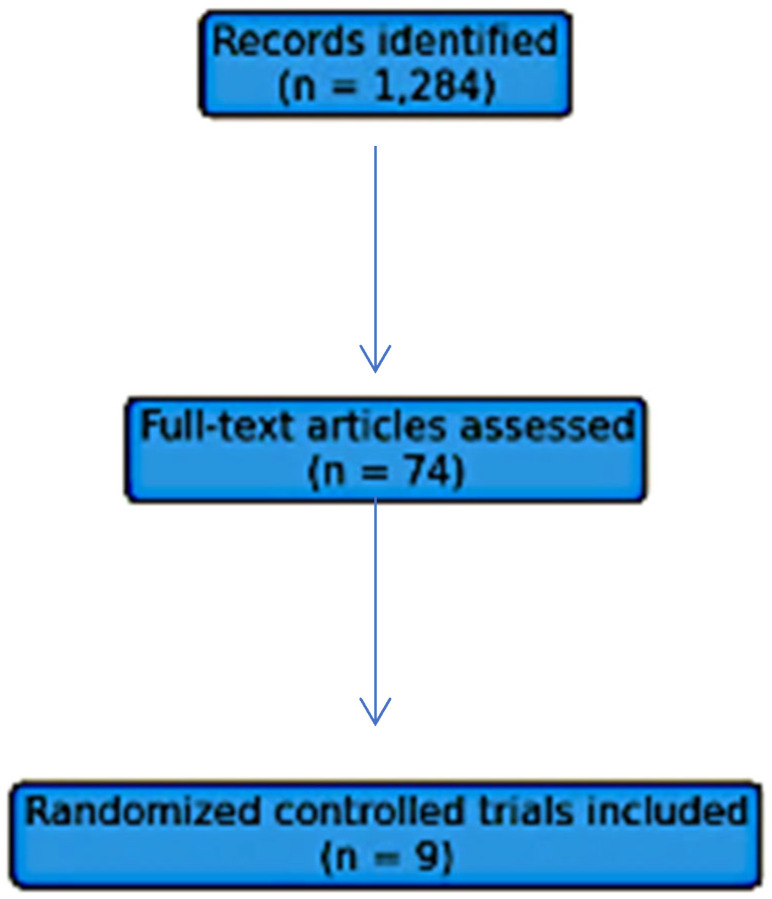
PRISMA flow diagram of study selection.

The systematic literature search identified 1284 records after duplicate removal. Following title and abstract screening, 74 full-text articles were assessed for eligibility. Nine randomized controlled trials met all predefined inclusion criteria and were included in the comparative synthesis of relapsing forms of multiple sclerosis. The study selection process is illustrated in Figure 1. Of the 74 full-text articles assessed, 65 were excluded. The reasons for exclusion included: study design not a parallel-group randomized controlled trial (e.g., open-label extension, post hoc analysis, single-arm study); duplicate publication or pooled analysis without unique outcome data; follow-up duration < 48 weeks; population not exclusively relapsing MS (e.g., mixed progressive cohorts without subgroup data); intervention not a monoclonal antibody of interest; comparator not eligible; or outcome definitions not meeting inclusion criteria (e.g., 3-month confirmed disability progression only). A complete list of excluded studies with full reasons for exclusion is available from the corresponding author upon reasonable request.

Across the included trials, a total of 6762 patients were randomized. Five monoclonal antibody therapies were evaluated: natalizumab, alemtuzumab, ocrelizumab, ofatumumab, and ublituximab. These agents were studied against placebo or active disease-modifying therapies, most commonly interferon beta-1a or teriflunomide. All included trials were phase III, parallel-group randomized controlled trials with follow-up durations ranging from 96 weeks to 30 months.

Trials enrolling exclusively patients with primary progressive multiple sclerosis were excluded from the primary comparative analyses due to fundamental differences in disease biology and outcome behavior and were evaluated separately in descriptive sensitivity analyses, Table 1.

The figure illustrates the identification, screening, eligibility assessment, and final inclusion of randomized controlled trials evaluating monoclonal antibody therapies in relapsing multiple sclerosis.

Risk of Bias Assessment. Risk of bias was assessed for all included randomized controlled trials using the Cochrane Risk of Bias 2.0 tool. Overall, the majority of trials were judged to be at low risk of bias across most domains. The randomization process was consistently assessed as low risk, with all pivotal phase III trials reporting adequate sequence generation and allocation concealment, Table 2 and Table 3.

#### Excluded Studies

Of the 74 full-text articles assessed for eligibility, 65 were excluded. The most frequent reasons for exclusion were: study design not a parallel-group randomized controlled trial (e.g., open-label extension, post hoc analysis, single-arm study); duplicate publication or pooled analysis without unique outcome data; follow-up duration <48 weeks; population not exclusively relapsing MS (e.g., mixed progressive cohorts without subgroup data); intervention not a monoclonal antibody of interest; comparator not eligible; outcome definitions not meeting inclusion criteria (e.g., 3-month confirmed disability progression only).

### 3.2. Network Geometry and Comparator-Defined Evidence Components

Network geometry was assessed separately for annualized relapse rate (ARR) and six-month confirmed disability progression (CDP), in accordance with the prespecified analytical framework.

For ARR, the randomized evidence base formed three disconnected comparator-defined components:

- A placebo-controlled component including natalizumab;
- An interferon beta-1a-controlled component including alemtuzumab and ocrelizumab;
- A teriflunomide-controlled component including ofatumumab and ublituximab.

No randomized links existed between these components, precluding indirect inference across comparator eras. For CDP, network connectivity was further reduced due to heterogeneity in disability outcome definitions and confirmation procedures. Alemtuzumab and ocrelizumab formed an interferon beta-1a-based component, while ofatumumab and ublituximab formed a teriflunomide-based component. Natalizumab was isolated for this outcome.

**Figure 2:**
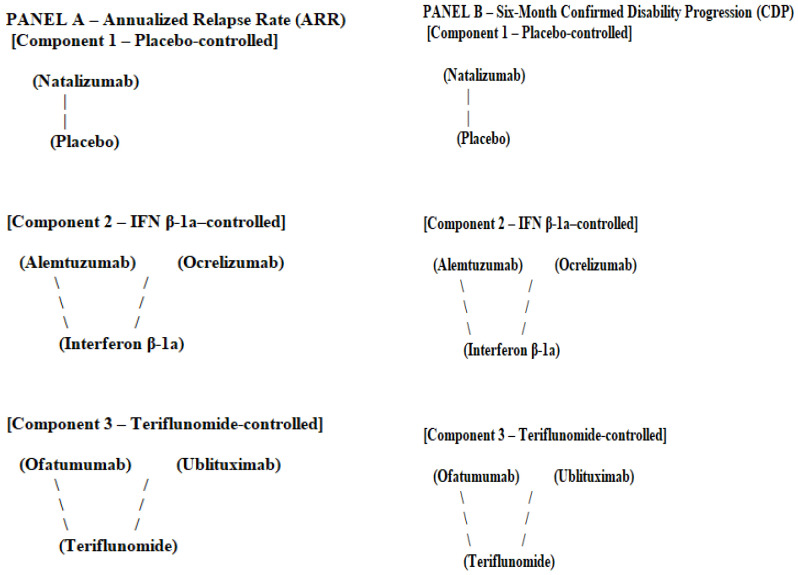
Network geometry for annualized relapse rate (ARR) and six-month confirmed disability progression (CDP). Legend: nodes represent randomized interventions and comparators; edges represent direct randomized comparisons within individual trials. For both outcomes, the evidence base consists of three fully disconnected comparator-defined components: (1) placebo-controlled (natalizumab vs. placebo), (2) interferon beta-1a-controlled (alemtuzumab and ocrelizumab vs. interferon beta-1a), and (3) teriflunomide-controlled (ofatumumab and ublituximab vs. teriflunomide). No direct or indirect links exist between components. Accordingly, indirect comparisons across components are not supported, and no global treatment hierarchy can be inferred.

Given these structural characteristics, all analyses were restricted to within-component comparisons. No cross-component indirect comparisons or global treatment hierarchies were estimated, Figure 2.

For ARR, the evidence base is fragmented into three disconnected comparator-defined components: a placebo-controlled component including natalizumab, an interferon beta-1a-controlled component including alemtuzumab and ocrelizumab, and a teriflunomide-controlled component including ofatumumab and ublituximab. No randomized links exist between these components, precluding indirect inference across comparator eras.

For six-month CDP, network connectivity is further reduced due to heterogeneity in outcome definitions and confirmation procedures. Alemtuzumab and ocrelizumab form an interferon beta-1a-based component, ofatumumab and ublituximab form a teriflunomide-based component, and natalizumab is isolated for this outcome.

### 3.3. Annualized Relapse Rate

All included monoclonal antibody therapies demonstrated substantial reductions in annualized relapse rate compared with their respective randomized comparators.

Footnote: Estimates are presented stratified by comparator-defined component. Direct comparisons of effect magnitudes across different components (e.g., natalizumab vs. ocrelizumab) are not supported by the available randomized evidence due to differences in baseline relapse risk, trial populations, and calendar periods.

This qualitative similarity suggests that, within the shared comparator framework of interferon beta-1a, both agents demonstrate large-magnitude effects relative to the same control. However, this observation does not constitute a comparative efficacy claim between alemtuzumab and ocrelizumab, nor does it imply equivalence. Formal indirect comparison was not performed, and cross-trial differences in baseline relapse rate, disease duration, and prior treatment exposure preclude any direct comparative interpretation. For ocrelizumab, ofatumumab, and ublituximab, each was evaluated in two identically designed phase III trials (OPERA I/II, ASCLEPIOS I/II, and ULTIMATE I/II, respectively). Individual trial results were consistent, with no evidence of heterogeneity (I^2^ = 0% for all pairs). Pooled fixed-effect estimates were therefore calculated and are presented in Table 3. Individual trial-level rate ratios were as follows: OPERA I: 0.53 (0.40–0.71); OPERA II: 0.55 (0.41–0.73); ASCLEPIOS I: 0.44 (0.32–0.60); ASCLEPIOS II: 0.48 (0.35–0.66); ULTIMATE I: 0.44 (0.29–0.67); ULTIMATE II: 0.46 (0.30–0.70).

Within the interferon beta-1a-controlled component, both alemtuzumab and ocrelizumab were associated with significantly lower ARR compared with interferon beta-1a. The rate ratios for alemtuzumab (0.47; 95% CI 0.34–0.65) and ocrelizumab (0.54; 95% CI 0.41–0.71) were of similar magnitude, and their confidence intervals showed substantial overlap. This qualitative similarity suggests broadly comparable relapse suppression, although formal indirect comparison was not performed.

### 3.4. Six-Month Confirmed Disability Progression

Six-month confirmed disability progression was reported in most included trials, although outcome definitions and confirmation procedures varied across studies, Table 4. For six-month confirmed disability progression, pooled hazard ratios were calculated for ocrelizumab (OPERA I/II) and ofatumumab (ASCLEPIOS I/II) using fixed-effect meta-analysis, as no heterogeneity was detected. For ublituximab, the two ULTIMATE trials provided individual estimates of 0.72 (0.40–1.29) and 0.87 (0.48–1.58); the pooled estimate was 0.79 (0.51–1.23) with no evidence of heterogeneity. Alemtuzumab (CARE-MS I and CARE-MS II) and natalizumab (AFFIRM) are presented as individual trial estimates without pooling, as these trials enrolled distinct populations (treatment-naïve vs. previously treated for alemtuzumab; no replicate trial for natalizumab); (Table 5).

Within the interferon beta-1a--based component, both ocrelizumab (HR 0.60; 95% CI 0.43–0.84) and alemtuzumab (HR 0.58; 95% CI 0.38–0.87) demonstrated statistically significant reductions in the risk of six-month confirmed disability progression compared with interferon beta-1a. The effect estimates were of similar magnitude and their confidence intervals showed substantial overlap. No indirect comparisons were conducted. Due to the absence of a common comparator network and heterogeneity in disability outcome definitions, all reported effect estimates represent direct comparisons from individual randomized controlled trials.

Of the nine included trials, six reported six-month CDP hazard ratios directly in the primary publication or its appendix. For the two alemtuzumab trials (CARE-MS I and CARE-MS II), six-month CDP hazard ratios were not directly reported and were derived using the Tierney et al. 2007 method [24], as described in Section 2.6. No other trials required derivation.

Importantly, the hazard ratios for six-month confirmed disability progression in the alemtuzumab trials were derived from reconstructed survival data and are therefore subject to greater uncertainty than directly reported estimates. These results should be interpreted with appropriate caution.

### 3.5. Transitivity Assessment and Clinical Heterogeneity

Assessment of potential effect modifiers revealed substantial heterogeneity across comparator-defined components. Differences were observed in baseline EDSS, baseline ARR, disease duration, prior treatment exposure, MRI activity, and calendar period of trial conduct.

Trials conducted in earlier comparator eras generally enrolled patients with higher inflammatory activity and lower prior treatment exposure, whereas more recent trials reflected evolving eligibility criteria and background therapy landscapes. These imbalances supported the restriction of indirect inference to within-component analyses and the avoidance of cross-component synthesis, Table 5.

#### 3.5.1. Assessment of Within-Component Comparability

Table 6 presents baseline demographic and disease characteristics for each individual trial, stratified by comparator component. This detailed disaggregation allows a more nuanced assessment of whether trials sharing a common comparator are sufficiently similar to support qualitative comparisons of treatment effects.

Within the interferon beta-1a component, some heterogeneity is present. The CARE-MS I trial enrolled exclusively treatment-naïve patients with short disease duration and high relapse activity, whereas CARE-MS II enrolled patients with a prior inadequate response to disease-modifying therapy. The OPERA trials enrolled a mixed population, with approximately 40% previously treated. Baseline EDSS and disease duration were slightly higher in OPERA than in CARE-MS I, but comparable to CARE-MS II. Despite these differences, all four trials enrolled patients with active relapsing MS and demonstrated consistent, large-magnitude treatment effects on relapse rate. For disability progression, treatment effects were also directionally consistent and of similar magnitude across trials. Therefore, while the populations are not identical, the consistency of effect sizes across these heterogeneous subgroups supports the interpretation that both alemtuzumab and ocrelizumab are highly effective relative to interferon beta-1a, and that qualitative comparison of their effect estimates is reasonable.

Within the teriflunomide component, the ASCLEPIOS and ULTIMATE trials are highly homogeneous. All four trials enrolled patients with active relapsing MS, with similar age, disease duration, baseline EDSS, prior treatment exposure, and MRI activity. The entry criteria and outcome definitions were nearly identical. This high degree of similarity supports the validity of comparing the magnitude of treatment effects between ofatumumab and ublituximab.

#### 3.5.2. Implications for Interpretation

In summary, the interferon beta-1a component contains moderate clinical heterogeneity, but the consistency of treatment effects across trials suggests that this does not undermine the qualitative comparison of alemtuzumab and ocrelizumab. The teriflunomide component is highly homogeneous, supporting more confident comparative interpretation. These assessments are necessarily qualitative and should be considered when drawing conclusions from the available evidence.

### 3.6. Risk of Bias Across Included Trials

Risk of bias assessment using the Cochrane Risk of Bias 2.0 tool indicated that the majority of included trials were at low risk of bias across key methodological domains, including randomization, deviations from intended interventions, and missing outcome data.

Some concerns were identified for the outcome measurement domain in several trials, primarily reflecting heterogeneity in disability progression definitions and confirmation procedures. These were interpreted as sources of indirectness rather than true outcome measurement bias. No evidence of selective reporting was identified.

### 3.7. Sensitivity Analyses

Sensitivity analyses restricted to phase III trials and excluding studies with higher risk of bias yielded results consistent with the primary analyses for both ARR and CDP.

Descriptive analyses of trials conducted exclusively in primary progressive multiple sclerosis further supported their exclusion from the primary comparative framework due to distinct disease characteristics and outcome behavior.

### 3.8. Summary of Results

Across comparator-defined connected evidence components, monoclonal antibody therapies demonstrated robust and consistent efficacy in reducing relapse activity relative to their respective comparators. Disability outcomes were more heterogeneous and context-dependent.

Owing to the fragmented structure of the randomized evidence base, comparative conclusions were limited to within-component analyses, and no global efficacy hierarchy was established.

## 4. Discussion

The present study provides a systematic and methodologically constrained synthesis of randomized evidence evaluating monoclonal antibody therapies in relapsing forms of multiple sclerosis. By presenting direct treatment effects stratified by shared randomized comparators and by formally accounting for network disconnection, comparator drift, and outcome heterogeneity, this work addresses several key methodological limitations that have affected prior comparative analyses in this therapeutic area [21,22,23].

It is important to emphasize that this study does not report a conventional network meta-analysis. Rather, it provides a comparator-stratified synthesis that respects the disconnected structure of the evidence base. Readers expecting global treatment rankings or indirect effect estimates across all agents will not find them here, as such analyses would require unsupported methodological assumptions.

A central finding of this study is that the available randomized evidence base does not support a fully connected comparative network for monoclonal antibody therapies in relapsing multiple sclerosis. Instead, pivotal trials form distinct comparator-era components defined by placebo, interferon beta-1a, or teriflunomide control arms. This structure reflects the historical evolution of MS clinical trial design, in which successive therapies were evaluated against changing standards of care rather than against previously approved high-efficacy agents [4,5,6,7,12,25]. Rather than attempting to overcome this limitation through unsupported indirect inference, the present analysis embraces this structure and restricts comparative conclusions to within-component analyses, thereby preserving the core assumptions of indirect comparison methodology [21,22,23].

These findings support the concept that, within each comparator-defined component, monoclonal antibody therapies consistently achieve substantial relapse reductions relative to their specific control. However, this observation should not be misinterpreted as evidence of comparable efficacy across components, given substantial differences in baseline relapse risk, trial eligibility criteria, and calendar periods. The term efficacy plateau is therefore applicable only within, not across, comparator-defined frameworks [5,6,25]. Similarly, in teriflunomide-controlled trials, ofatumumab and ublituximab demonstrated substantial and broadly comparable reductions in relapse activity [7,12]. Descriptively, the magnitude of relapse reduction appears broadly similar across agents when evaluated within the same comparator framework. However, this observation should be interpreted cautiously: overlapping confidence intervals do not demonstrate equivalence, and formal statistical comparison for non-inferiority was not performed. The term ‘efficacy plateau’ is therefore used here as a descriptive observation, not as a statistically tested claim.

The analysis of 6-month confirmed disability progression is appropriately cautious in the methods, but the results and discussion still risk overinterpretation. Given the heterogeneity of definitions, limited event counts, and wide confidence intervals, the authors should consider whether these findings are best presented as demonstrating the limits of current evidence rather than as comparative efficacy signals. [4,5,6,7,12,25].

The analysis of 6-month confirmed disability progression is appropriately cautious in the methods, but the results and discussion still risk overinterpretation. Given the heterogeneity of definitions, limited event counts, and wide confidence intervals, we consider these findings to demonstrate the limits of current evidence rather than to provide reliable comparative efficacy signals. This heterogeneity likely reflects a combination of factors, including differences in baseline disability, disease duration, prior treatment exposure, follow-up duration, and, critically, variability in disability outcome definitions and confirmation procedures across trials [26,27,28]. Importantly, such variability was interpreted in this study as a source of indirectness rather than as a manifestation of methodological bias, underscoring the need for cautious interpretation of disability-related comparative findings.

A key feature of this analysis is the deliberate avoidance of global treatment ranking and hierarchy estimation. Given the disconnected structure of the evidence base, ranking metrics such as SUCRA would not be statistically supported and could be clinically misleading. By refraining from imposing a global hierarchy and instead presenting comparator-anchored effect estimates, we prioritize methodological validity and interpretability.

Beyond its substantive findings, this study illustrates an approach to evidence synthesis in therapeutic areas characterized by comparator drift and network fragmentation. The analytical framework applied here explicit pre-specification of network connectivity requirements, restriction of inference to comparator-defined components, structured transitivity assessment can inform future systematic reviews and health technology assessments facing similar structural constraints [21,22,23,29].

Beyond its substantive findings, this study advances the field by demonstrating a generalizable methodological approach for evidence synthesis in therapeutic areas characterized by comparator drift and network fragmentation. The analytical framework applied here explicit pre-specification of network connectivity requirements, restriction of inference to comparator-defined components, structured transitivity assessment, and refusal to generate unsupported global rankings can and should be adopted by future systematic reviews, health technology assessments, and clinical guideline development processes. In this regard, the present work serves as a methodological corrective, illustrating that rigorous evidence synthesis sometimes requires accepting the limits of available data rather than attempting to overcome them through statistical imputation or model-based assumptions.

The structured assessment of transitivity further supports this conservative analytical approach. Substantial imbalances were observed across comparator-defined components in baseline disease characteristics, including EDSS, annualized relapse rate, disease duration, MRI activity, and prior treatment exposure. These differences reflect evolving eligibility criteria and shifting therapeutic landscapes over time and reinforce the limitations of cross-era indirect inference [21,22,23,27,28,29]. The explicit identification of these imbalances strengthens the rationale for restricting comparative conclusions to trials sharing a common randomized comparator.

From a clinical perspective, the findings of this study suggest that, within comparable trial frameworks, multiple monoclonal antibody therapies provide similarly robust control of inflammatory disease activity. In the absence of clear relapse-related efficacy superiority among agents, treatment selection in clinical practice should be guided by individualized considerations rather than assumptions of global comparative advantage. These considerations include baseline disease activity, risk of disability accumulation, safety profile, monitoring requirements, route and frequency of administration, comorbidities, and patient preferences [27,28,29].

Safety considerations, while not quantitatively synthesized in this analysis due to heterogeneity in reporting and follow-up, remain central to therapeutic decision-making for monoclonal antibody therapies. Differences in risks of serious infections, infusion-related reactions, secondary autoimmunity, hypogammaglobulinemia, and rare opportunistic infections such as progressive multifocal leukoencephalopathy may meaningfully influence treatment choice, particularly in long-term disease management [4,5,6,7,12,25]. The absence of a global efficacy hierarchy further underscores the importance of integrating safety and tolerability considerations into individualized treatment strategies.

Several limitations of this study merit consideration. Restriction of comparative analyses to comparator-defined components limits the ability to draw broad cross-class comparisons between monoclonal antibodies evaluated against different comparators; however, this limitation reflects the structure of the available randomized evidence rather than a deficiency of the analytical approach [21,22,23]. Additionally, despite predefined sensitivity analyses and structured assessment of effect modifiers, residual confounding due to unmeasured trial-level characteristics cannot be fully excluded. Finally, heterogeneity in disability outcome definitions necessitated cautious interpretation of disability-related findings [26,27,28].

In particular, the analysis of six-month confirmed disability progression should be viewed not as a source of comparative efficacy estimates, but as a demonstration of the need for harmonized outcome definitions and larger, adequately powered trials designed specifically to detect differences in disability accumulation [21,22,23,29].

It is important to clarify the descriptive nature of comparisons suggesting similar efficacy across agents. Overlapping confidence intervals between independent trial estimates do not demonstrate equivalence or non-inferiority. Formal demonstration of comparable efficacy would require dedicated non-inferiority trials with prespecified margins, which have not been conducted for these agents. Therefore, observations of similar effect magnitudes should be interpreted as descriptive findings that generate hypotheses, not as confirmatory evidence of equivalent therapeutic effect.

### 4.1. Clinical and Guideline Implications of Within-Component Comparisons

This study enables three categories of clinically actionable inferences. First, justified inferences: within the interferon beta-1a component, both alemtuzumab and ocrelizumab provide comparable and substantial relapse reduction, and both reduce six-month disability progression relative to interferon beta-1a. Within the teriflunomide component, ofatumumab and ublituximab provide comparable and substantial relapse reduction; disability benefit is statistically significant for ofatumumab, while for ublituximab the confidence interval includes the null, though the point estimate is consistent with a modest effect. These anchored comparisons represent the highest-quality available evidence and can directly inform treatment guidelines and shared decision-making when choosing among agents evaluated against the same comparator.

Second, unsupported inferences: no valid indirect comparison can be made across comparator-defined components (e.g., natalizumab vs. ocrelizumab, or ofatumumab vs. alemtuzumab) due to network disconnection and non-transitivity. Any suggestion of global superiority or ranking across these agents is not supported by randomized evidence. Similarly, no conclusions can be drawn regarding comparative disability outcomes across components, given heterogeneity in outcome definitions and patient populations.

Third, implications for interpretation of prior comparative studies: many published network meta-analyses and global treatment rankings (e.g., SUCRA) have implicitly assumed full network connectivity and transitivity, producing hierarchies that are statistically unsupported and potentially misleading. This study demonstrates that such rankings are not reproducible when methodological constraints are rigorously applied. Clinicians and guideline developers should therefore view existing comparative effectiveness claims with caution and prioritize comparator-anchored evidence over global hierarchies. Future research should adopt shared active comparators and harmonize disability endpoints to enable valid comparative inference.

Additionally, for the alemtuzumab trials, six-month confirmed disability progression hazard ratios were not directly reported and were derived using reconstruction methods from published survival curves. Although we applied a validated and widely accepted methodology [24], this approach introduces additional uncertainty compared to directly reported treatment effects, particularly regarding the proportional hazards assumption and the accuracy of curve digitization. Sensitivity analyses excluding the alemtuzumab trials did not materially alter the conclusions regarding disability progression within the interferon beta-1a component.

### 4.2. Safety Considerations in Context

Although comparative efficacy is a central consideration in treatment selection, safety and tolerability profiles are equally critical for informed clinical decision-making. The qualitative summary of safety outcomes presented in Table 6 highlights important differences among monoclonal antibody therapies that are not captured by efficacy comparisons alone. Alemtuzumab, while highly effective, carries a well-documented risk of secondary autoimmune adverse events, necessitating long-term monitoring. Natalizumab is associated with PML risk, which requires risk stratification and vigilance. Anti-CD20 therapies (ocrelizumab, ofatumumab, ublituximab) have favorable safety profiles overall, with infusion- or injection-related reactions being the most frequent adverse events and infection risks that are modestly increased but generally manageable. These safety considerations, together with patient preferences, comorbidities, and lifestyle factors, should inform individualized treatment decisions alongside the comparative efficacy data presented in this review.

## 5. Conclusions

This systematic review provides a comparator-stratified synthesis of monoclonal antibody therapies in relapsing MS, rather than a fully connected network meta-analysis. By explicitly assessing network geometry, comparator-era structure, and outcome-specific connectivity prior to quantitative synthesis, the present study addresses key methodological challenges inherent to comparative effectiveness research in multiple sclerosis. Furthermore, apparent similarities in relapse rate ratios across different comparator components should not be interpreted as evidence of comparable absolute effectiveness, given the substantial differences in baseline relapse risk and trial populations. Similarly, the disability progression findings for alemtuzumab are derived from reconstructed data and carry greater uncertainty than directly reported estimates, a distinction that must be acknowledged in any comparative interpretation.

The findings demonstrate that monoclonal antibody therapies consistently reduce relapse activity relative to their respective randomized comparators within comparator-defined connected components. When evaluated within comparable trial frameworks, treatment effects on annualized relapse rate appear broadly similar across high-efficacy agents in descriptive terms. This observation suggests that all evaluated monoclonal antibodies are highly effective relative to their respective comparators, but it does not constitute statistical evidence of equivalent efficacy. Formal comparisons of equivalence or non-inferiority would require dedicated trial designs.

In contrast, effects on six-month confirmed disability progression were more heterogeneous and context-dependent. Differences in trial design, baseline patient characteristics, and disability outcome definitions limit the interpretability of cross-trial comparisons and underscore the need for cautious interpretation of disability-related efficacy outcomes. These findings highlight the importance of contextualizing disability outcomes within the specific experimental frameworks in which they were generated.

This systematic review provides a methodologically rigorous framework for interpreting comparative efficacy of monoclonal antibody therapies in relapsing multiple sclerosis. The findings demonstrate three key principles for clinical practice and guideline development.

First, within comparator-defined component where direct randomized evidence exists multiple monoclonal antibodies show broadly comparable and substantial reductions in relapse activity.

Second, no valid inferences can be drawn across components. Comparisons between agents evaluated against different controls (e.g., natalizumab vs. ocrelizumab, or alemtuzumab vs. ofatumumab) are not supported by the available randomized evidence. Global treatment rankings that suggest superiority across such agents are methodologically invalid and clinically misleading. In contrast, for six-month confirmed disability progression, the evidence is characterized by substantial uncertainty. Point estimates vary, confidence intervals are wide, and differences in outcome definitions and confirmation procedures limit interpretability. The primary contribution of the disability analysis is therefore to demonstrate the insufficiency of current randomized evidence for informing comparative judgments on disability progression rather than to provide reliable estimates of treatment efficacy.

Third, this work directly challenges the interpretation of many existing comparative studies and network meta-analyses that have reported comprehensive efficacy hierarchies. By demonstrating that such hierarchies are not reproducible under rigorous methodological constraints, the present study provides a strong rationale for guideline developers to prioritize anchored, comparator-specific evidence and to avoid reliance on unsupported indirect comparisons.

In conclusion, this study provides a systematic synthesis of randomized evidence on monoclonal antibody therapies in relapsing MS, with explicit attention given to network connectivity and comparator heterogeneity. The core contribution is the application of a disciplined analytical framework that avoids unsupported indirect comparisons and instead presents comparator-anchored effect estimates.

Methodologically, the study demonstrates the importance of transparency about what cannot be estimated, given the structural constraints of the evidence base. For clinical practice and guideline development, it offers an explicit distinction between justified within-component comparisons and unsupported cross-component inferences. For future research, it highlights the need for shared active comparators and harmonized disability outcome definitions to enable valid comparative inference.

By reframing the question from “which therapy is superior?” to “what can validly be concluded from the available evidence?”, this analysis aims to support more informed treatment decisions and more rigorous trial design in multiple sclerosis research.

## Figures and Tables

**Table 1:** Risk of bias assessment of included randomized controlled trials using the Cochrane Risk of Bias 2.0 tool.

Study	Randomization Process	Deviations from Intended Interventions	Missing Outcome Data	Outcome Measurement	Selection of Reported Results	Overall Risk of Bias
AFFIRM	Low	Low	Low	Low	Low	Low
CARE-MS I	Low	Low	Low	Some concerns	Low	Some concerns
CARE-MS II	Low	Low	Low	Some concerns	Low	Some concerns
OPERA I	Low	Low	Low	Low	Low	Low
OPERA II	Low	Low	Low	Low	Low	Low
ASCLEPIOS I	Low	Low	Low	Some concerns	Low	Some concerns
ASCLEPIOS II	Low	Low	Low	Some concerns	Low	Some concerns
ULTIMATE I	Low	Low	Low	Some concerns	Low	Some concerns
ULTIMATE II	Low	Low	Low	Some concerns	Low	Some concerns

Legend: Risk of bias was assessed across five domains according to the Cochrane Risk of Bias 2.0 tool. “Some concerns” for outcome measurement primarily reflect heterogeneity in disability progression definitions rather than deficiencies in trial conduct.

**Table 2:** Baseline demographic and disease characteristics of included trials, by comparator component.

Trial	Component	N	Mean/Median Age (Years)	Female (%)	Mean Disease Duration (Years)	Mean Baseline EDSS	Mean Baseline ARR (Prior Year)	Prior DMT Use (%)	Gd+ Lesions (%)
AFFIRM	Placebo	942	36	72	5.0	2.3	1.5	0%	54%
CARE-MS I	IFN β-1a	563	33	64	2.1	2.0	1.8	0%	48%
CARE-MS II	IFN β-1a	628	35	68	4.5	2.5	1.7	100%	43%
OPERA I	IFN β-1a	821	37	66	3.8	2.8	1.3	38%	40%
OPERA II	IFN β-1a	836	37	65	3.9	2.8	1.3	40%	38%
									
ASCLEPIOS I	Teriflunomide	927	38	70	5.1	2.9	1.1	55%	32%
ASCLEPIOS II	Teriflunomide	955	38	69	5.2	2.9	1.1	56%	33%
ULTIMATE I	Teriflunomide	545	37	68	5.5	3.0	1.2	60%	30%
ULTIMATE II	Teriflunomide	545	38	69	5.6	3.1	1.2	61%	31%

Note: Values are approximate, extracted from primary publications and appendix. DMT = disease-modifying therapy; Gd+ = gadolinium-enhancing; EDSS = Expanded Disability Status Scale; ARR = annualized relapse rate.

**Table 3:** Characteristics of randomized controlled trials included in the analysis.

Trial	Population	Intervention	Comparator	Total N	Follow-Up
AFFIRM	RRMS	Natalizumab	Placebo	942	24 months
CARE-MS I	RRMS (treatment-naïve)	Alemtuzumab	IFN β-1a	563	24 months
CARE-MS II	RRMS (previously treated)	Alemtuzumab	IFN β-1a	628	24 months
OPERA I	RRMS	Ocrelizumab	IFN β-1a	821	96 weeks
OPERA II	RRMS	Ocrelizumab	IFN β-1a	836	96 weeks
ASCLEPIOS I	RRMS	Ofatumumab	Teriflunomide	927	30 months
ASCLEPIOS II	RRMS	Ofatumumab	Teriflunomide	955	30 months
ULTIMATE I	RRMS	Ublituximab	Teriflunomide	545	96 weeks
ULTIMATE II	RRMS	Ublituximab	Teriflunomide	545	96 weeks

**Table 4:** Six-Month Confirmed Disability Progression (CDP)—Hazard Ratios by Comparator-Controlled Components.

Component	Treatment	Comparator	Hazard Ratio	95% CI
Placebo-controlled	Natalizumab	Placebo	0.58	0.43–0.77
Interferon beta-1a-controlled	Alemtuzumab	IFN β-1a	0.58	0.38–0.87
	Ocrelizumab	IFN β-1a	0.60	0.43–0.84
Teriflunomide-controlled	Ofatumumab	Teriflunomide	0.67	0.48–0.97
	Ublituximab	Teriflunomide	0.79	0.51–1.23

**Table 5:** Assessment of transitivity across comparator-defined evidence components.

Effect Modifier	Placebo-Controlled Trials (Natalizumab)	IFN β-1a-Controlled Trials (Alemtuzumab, Ocrelizumab)	Teriflunomide-Controlled Trials (Ofatumumab, Ublituximab)
**Baseline EDSS**	~2.0–3.0	~2.0–3.5	~2.5–4.0
**Baseline ARR**	High (≥1.5–2.0)	Moderate–high (~1.3–1.8)	Moderate (~1.1–1.5)
**Disease duration**	Short–moderate	Moderate	Longer
**Prior DMT exposure**	Minimal/treatment-naïve predominant	Mixed (naïve + previously treated)	Majority previously treated
**Baseline MRI activity**	High lesion burden and Gd+ activity	Moderate–high	Moderate
**Calendar period**	Early comparator era	Transitional era	Recent trials
**Eligibility criteria stringency**	Less restrictive	Intermediate	More restrictive

Abbreviations: DMT, disease-modifying therapy; EDSS, Expanded Disability Status Scale; Gd+, gadolinium-enhancing lesions.

**Table 6:** Summary of key safety outcomes from included randomized controlled trials (controlled treatment period).

**Trial**	**Intervention**	**Comparator**	**SAEs (%)**	**D/C Due to AEs (%)**	**Infusion/Injection Reactions (%)**	**Serious Infections (%)**	**Notable Specific AEs**
**AFFIRM**	Natalizumab	Placebo	19.0 vs. 18.0	6.0 vs. 4.0	24.0 vs. 18.0 (any IRR)	3.2 vs. 2.6	PML: 1 case (0.1%)
**CARE-MS I**	Alemtuzumab	IFN β-1a	18.0 vs. 14.0	6.0 vs. 4.0	90.0 vs. 21.0 (any IRR)	2.0 vs. 2.0	Thyroid AEs: 18.0% vs. 6.0%; ITP: <1%
**CARE-MS II**	Alemtuzumab	IFN β-1a	21.0 vs. 16.0	7.0 vs. 6.0	87.0 vs. 23.0 (any IRR)	4.0 vs. 1.0	Thyroid AEs: 16.0% vs. 5.0%; ITP: <1%
**OPERA I**	Ocrelizumab	IFN β-1a	22.0 vs. 23.0	7.0 vs. 8.0	34.0 vs. 10.0 (any IRR)	1.3 vs. 2.9	No PML; any infection: 58.0% vs. 52.0%
**OPERA II**	Ocrelizumab	IFN β-1a	21.0 vs. 22.0	6.0 vs. 8.0	34.0 vs. 10.0 (any IRR)	1.5 vs. 1.9	No PML; any infection: 60.0% vs. 55.0%
**ASCLEPIOS I**	Ofatumumab	Teriflunomide	11.0 vs. 12.0	7.0 vs. 8.0	21.0 vs. 16.0 (local injection-related)	1.7 vs. 1.9	No PML; any infection: 51.0% vs. 49.0%
**ASCLEPIOS II**	Ofatumumab	Teriflunomide	12.0 vs. 13.0	8.0 vs. 9.0	20.0 vs. 15.0 (local injection-related)	1.8 vs. 2.0	No PML; any infection: 52.0% vs. 50.0%
**ULTIMATE I**	Ublituximab	Teriflunomide	12.0 vs. 13.0	6.0 vs. 7.0	48.0 vs. 15.0 (any IRR)	1.8 vs. 1.9	No PML
**ULTIMATE II**	Ublituximab	Teriflunomide	11.0 vs. 12.0	5.0 vs. 7.0	48.0 vs. 15.0 (any IRR)	1.8 vs. 1.9	No PML

Abbreviations: SAEs = serious adverse events; D/C due to AEs = discontinuation due to adverse events; IRR = infusion-related reaction; PML = progressive multifocal leukoencephalopathy; ITP = immune thrombocytopenia; AE = adverse event; IFN = interferon; vs. = versus.

## Data Availability

The original contributions presented in this study are included in the article/Supplementary Materials. Further inquiries can be directed to the corresponding author.

## Supplementary Materials

The following supporting information can be downloaded at: https://www.mdpi.com/article/10.3390/medsci14010116/s1, Table S1. Full-Text Articles Excluded After Eligibility Assessment (n = 65).

## Appendix A

**Table A1:** Summary of Efficacy Estimates Included in the Analysis (Rate Ratio and 95% Confidence Interval).

Intervention	Comparator	Rate Ratio	95% Confidence Interval
Natalizumab	Placebo	0.34	0.27–0.42
Alemtuzumab	IFN β-1a	0.47	0.34–0.65
Ocrelizumab	IFN β-1a	0.54	0.41–0.71
Ofatumumab	Teriflunomide	0.46	0.36–0.59
Ublituximab	Teriflunomide	0.45	0.33–0.62

The Rate Ratio values for Ofatumumab and Ublituximab were derived from pooled analyses of the ASCLEPIOS I & II and ULTIMATE I & II trials, respectively. These data demonstrate a significant reduction in annualized relapse rate (ARR) with anti-CD20 monoclonal antibodies compared with Teriflunomide.

This figure displays rate ratios and 95% confidence intervals for each monoclonal antibody therapy compared with its respective randomized control. Estimates are grouped by shared comparator to reflect the disconnected structure of the evidence base (see Figure 2).

IMPORTANT: Direct comparisons of effect magnitudes across different comparator components (e.g., natalizumab vs. ocrelizumab, or ofatumumab vs. alemtuzumab) are NOT supported by the available randomized evidence. Differences in baseline relapse risk, trial populations, disease duration, prior treatment exposure, and calendar periods preclude valid indirect inference across components. The visual alignment of estimates in this figure is for descriptive purposes only and does not imply network connectivity or transitivity.

Each square represents the point estimate; horizontal lines represent 95% confidence intervals. The vertical dashed line (RR = 1) indicates no treatment effect. All estimates shown are statistically significant at α = 0.05.

Pooled estimates are shown for ocrelizumab (OPERA I/II), ofatumumab (ASCLEPIOS I/II), and ublituximab (ULTIMATE I/II).

This figure should not be used to infer comparative efficacy across different comparator-defined components. For justified within-component comparisons, see Section 3.3 and Section 4.

### Complete Search Strategies

Detailed Search Strategy

1. Information Sources

A systematic literature search was conducted in the following electronic databases:

PubMed/MEDLINE

Embase (via Elsevier)

Cochrane Central Register of Controlled Trials (CENTRAL)

The search covered all records from database inception to 30 April 2025.

No language restrictions were applied at the search stage.

Reference lists of included trials and relevant review articles were screened manually to identify additional eligible studies.

Conference abstracts, trial registries, and unpublished data were not systematically searched.

2. PubMed/MEDLINE Search Strategy

Search date: 30 April 2025

Search string:

(“multiple sclerosis” [ MeSH Terms ] OR “multiple sclerosis” [ Title/Abstract ] )

AND

(“monoclonal antibodies” [ MeSH Terms ]

 OR monoclonal antibody [ Title/Abstract ]

 OR natalizumab [ Title/Abstract ]

 OR alemtuzumab [ Title/Abstract ]

 OR ocrelizumab [ Title/Abstract ]

 OR ofatumumab [ Title/Abstract ]

 OR ublituximab [ Title/Abstract ])

AND

(“randomized controlled trial”[Publication Type]

 OR randomized [ Title/Abstract ]

 OR randomized [ Title/Abstract ]

 OR placebo [ Title/Abstract ] )

3. Embase Search Strategy (Elsevier interface)

Search date: 30 April 2025

(‘multiple sclerosis’/exp OR ‘multiple sclerosis’:ti,ab)

AND

(‘monoclonal antibody’/exp

 OR natalizumab:ti,ab

 OR alemtuzumab:ti,ab

 OR ocrelizumab:ti,ab

 OR ofatumumab:ti,ab

 OR ublituximab:ti,ab)

AND

(‘randomized controlled trial’/exp

 OR random*:ti,ab

 OR placebo*:ti,ab)

Limits applied:

Human studies

Adult population

Conference abstracts were excluded at screening stage if no full publication was available.

4. Cochrane Central Register of Controlled Trials (CENTRAL)

Search date: 30 April 2025

Search strategy:

(“multiple sclerosis”)

AND

(natalizumab OR alemtuzumab OR ocrelizumab OR ofatumumab OR ublituximab)

AND

(randomized OR randomised OR placebo)

5. Additional Screening

Reference lists of all included trials were screened manually.

Reference lists of relevant systematic reviews were examined.

Duplicate records were removed prior to screening.

Titles and abstracts were screened independently by two reviewers.

Full texts were assessed against predefined eligibility criteria.

6. Study Selection and Exclusion Documentation

A complete trial-by-trial list of excluded full-text articles with specific reasons for exclusion is provided in Supplementary Table S1, in accordance with PRISMA 2020 recommendations.
