# Supplementary material for: Systematic Review of Monoclonal Antibody Therapies in Relapsing Multiple Sclerosis: Comparator-Stratified Analysis of Relapse and Disability Outcomes
Source: Med Sci (Basel). 2026 Feb 27;14(1):116. doi: 10.3390/medsci14010116 (PMC13027482; doi:10.3390/medsci14010116)
Supplement: Supplementary file 1 [file medsci-14-00116-s001.zip › medsci-4144783-supplementary.pdf]

Supplementary Table S1. Full-Text Articles Excluded After Eligibility Assessment (n = 65)

First Author / Group	Year	Study Identifier (Short Title/Description)	Primary Reason for Exclusion
Krämer	2022	Narrative review on monoclonal antibodies	Study design – Narrative review (not RCT)
Rolfes	2020	Review of failed MS trials	Study design – Review article
Arnold	2016	Conference abstract	Study design – Conference abstract only
Arnold	2015	Conference abstract	Study design – Conference abstract only
Barkhof	2015	Conference abstract	Study design – Conference abstract only
Coles	2016	Conference abstract	Study design – Conference abstract only
Coles	2011	Conference abstract	Study design – Conference abstract only
Cree	2019	Conference abstract	Study design – Conference abstract only
Deshmukh	2019	Conference abstract	Study design – Conference abstract only
Diaz	2014	Editorial commentary	Study design – Editorial
Giovannoni	2020	Open-label extension study	Study design – Open-label extension
Giovannoni	2022	Open-label extension study	Study design – Open-label extension
Havrdova	2017	Post-hoc analysis	Study design – Post-hoc analysis
Coles	2015	Long-term follow-up analysis	Study design – Extension study
Bell Gorrod	2020	Treatment switching analysis	Study design – Secondary analysis
Cree	2021	Post-hoc analysis	Study design – Post-hoc analysis
Cree	2017	Post-hoc analysis	Study design – Post-hoc analysis
Cree	2019	Post-hoc analysis	Study design – Post-hoc analysis
Agius	2014	Mixed MS population study	Population – Not exclusively relapsing MS
Arroyo Gonzalez	2017	Mixed phenotype MS	Population – Not exclusively relapsing MS
Barkhof	2014	Progressive MS cohort	Population – Not exclusively relapsing MS
Bass	2021	Progressive subgroup	Population – Not exclusively relapsing MS

Butzkueven	2020	Mixed cohort	Population – Not exclusively relapsing MS
Chitnis	2014	Pediatric MS cohort	Population – Not adult relapsing MS
Cohen	2010	Mixed disease forms	Population – Not exclusively relapsing MS
Coles	2008	Early MS mixed phenotype	Population – Not exclusively relapsing MS
Coles	2011	Mixed phenotype MS	Population – Not exclusively relapsing MS
Comi	2017	Mixed phenotype	Population – Not exclusively relapsing MS
Giovannoni	2016	Progressive subgroup	Population – Not exclusively relapsing MS
Graves	2013	Mixed phenotype	Population – Not exclusively relapsing MS
Kappos	2011	Mixed MS population	Population – Not exclusively relapsing MS
Lublin	2016	Progressive MS cohort	Population – Not exclusively relapsing MS
Montalban	2017	Primary progressive MS trial	Population – PPMS only
O'Connor	2011	Mixed population	Population – Not exclusively relapsing MS
ATAMS Investigators	2014	Atacicept trial	Intervention – Not monoclonal antibody of interest
ATON Investigators	2015	Atacicept in optic neuritis	Intervention – Not monoclonal antibody of interest
Tabalumab Investigators	2018	Tabalumab trial	Intervention – Not approved mAb
ACCLAIM Investigators	2015	Abatacept trial	Intervention – Not monoclonal antibody of interest
SOLAR Investigators	2019	Vitamin D3 trial	Intervention – Not monoclonal antibody
CHANGE-MS Investigators	2018	Temelimab trial	Intervention – Not approved monoclonal antibody
INSPIRE Investigators	—	Raltegravir trial	Intervention – Not monoclonal antibody
Hartung	2015	Other biologic therapy	Intervention – Not monoclonal antibody of interest
Phase I (Novartis)	—	Early-phase trial	Follow-up duration < 48 weeks
Phase I (Biogen)	—	Early-phase trial	Follow-up duration < 48 weeks
Phase I (Genentech)	—	Early-phase trial	Follow-up duration < 48 weeks

Phase IIa (Merck)	—	Early-phase trial	Follow-up duration < 48 weeks
Phase IIa (Roche)	—	Early-phase trial	Follow-up duration < 48 weeks
Confavreux	2014	Disability outcome 3-month only	Outcome – No 6-month CDP reported
Freedman	2015	Disability outcome 3-month only	Outcome – No 6-month CDP reported
Hartung	2016	Disability outcome 3-month only	Outcome – No 6-month CDP reported
Hutchinson	2014	Disability outcome 3-month only	Outcome – No 6-month CDP reported
Kappos	2015	Disability outcome 3-month only	Outcome – No 6-month CDP reported
Miller	2014	Disability outcome 3-month only	Outcome – No 6-month CDP reported
Pozzilli	2015	Disability outcome 3-month only	Outcome – No 6-month CDP reported
Vermersch	2016	Disability outcome 3-month only	Outcome – No 6-month CDP reported
Balcer	2013	Duplicate publication	Duplicate publication
Comi	2017	Duplicate publication	Duplicate publication
Graves	2013	Duplicate publication	Duplicate publication
Hughes	2010	Duplicate publication	Duplicate publication
Hunter	2019	Duplicate publication	Duplicate publication
Selmaj	2017	Duplicate publication	Duplicate publication
ECTRIMS Congress Abstracts	2018	Multiple abstracts screened	Conference abstract – Insufficient full-text data
AAN Annual Meeting Abstracts	2019	Multiple abstracts screened	Conference abstract – Insufficient full-text data
ACTRIMS Forum Abstracts	2020	Multiple abstracts screened	Conference abstract – Insufficient full-text data
ECTRIMS Congress Abstracts	2021	Multiple abstracts screened	Conference abstract – Insufficient full-text data
